# Metabolic hijacking styles: a review of how viral life cycles dictate glucose metabolism reprogramming

**DOI:** 10.3389/fmicb.2025.1690133

**Published:** 2025-12-03

**Authors:** Ziyi Bie, Yixing Tan, Chun Ye, Ke Wei

**Affiliations:** 1 Medical School, Hunan University of Chinese Medicine, Changsha, China; 2 Department of Clinical Laboratory, Central Hospital of Yongzhou, Yongzhou, China

**Keywords:** viral infection, glucose metabolism, glycolytic enzymes, metabolic reprogramming, host–virus interaction

## Abstract

Viral infection profoundly reprograms host glucose metabolism to support replication. This review proposes a “Sprint vs. Marathon” framework to explain how viral life cycles shape distinct metabolic hijacking styles. Acute RNA viruses employ a rapid, high-intensity “Sprint” strategy, aggressively activating glycolysis through pathways such as PI3K/Akt and HIF-1α. In contrast, chronic and latent viruses adopt a sustained “Marathon” strategy, subtly modulating glycolytic enzymes, glucose transporters, and survival pathways including NF-κB and mTOR. Understanding these divergent metabolic programs provides new insight into viral pathogenesis and highlights opportunities for developing host-directed antiviral therapies.

## 1 Introduction

Metabolism is fundamental to cell survival and function, and signaling is essential for the regulation and coordination of cellular metabolism. Since viruses are obligate intracellular parasites with no metabolic capacity of their own, they must actively hijack host cellular machinery to replicate successfully. This process involves interfering with key signaling pathways to manipulate cellular energy and nutrient metabolism for their own benefit (Pant et al., 2021). During infection, viruses profoundly reprogram host metabolic networks, including glycolysis, amino acid synthesis, and nucleotide biosynthesis, to generate the energy (ATP) and biomolecular precursors required for viral replication (Girdhar et al., 2021).

This viral-induced metabolic reprogramming is now recognized as a hallmark of infection. However, a critical question remains largely unexplored: do all viruses hijack host metabolism in the same way? Growing evidence suggests that the strategies employed are not monolithic. Instead, they appear to be highly adapted, reflecting the unique evolutionary pressures dictated by a virus’s fundamental biology, particularly its life cycle (acute vs. chronic) and genome type (RNA vs. DNA). These adaptations result in distinct “metabolic hijacking styles” that are intrinsically linked to viral pathogenesis.

This review aims to synthesize recent findings through this novel analytical lens. We propose a framework that categorizes these strategies into two major styles: a “Sprint” (or “Blitzkrieg”) style, characteristic of many acute RNA viruses, and a “Marathon” style, often employed by chronic or latent DNA viruses.

The “Sprint” style is defined by a rapid, aggressive, and often inefficient reprogramming of host metabolism, prioritizing maximum viral yield in the shortest possible time. Viruses like influenza A virus (IAV) exemplify this approach. They trigger a dramatic upregulation of aerobic glycolysis, often at the expense of inducing a strong pro-inflammatory response and significant host cell damage, a strategy well-suited for a “hit-and-run” life cycle (Thyrsted and Holm, 2021).

In contrast, the “Marathon” style involves a more subtle, sophisticated, and sustainable manipulation of the host cell. Viruses that establish chronic or latent infections, such as human cytomegalovirus (HCMV) or hepatitis B virus (HBV), must ensure the long-term survival and stability of their host cell reservoir. Their metabolic hijacking is therefore geared not just toward replication, but also toward promoting cell survival, inhibiting apoptosis, and modulating the immune response to facilitate persistent infection (Munger et al., 2006; Wang and Zhang, 2023).

By examining a range of representative viruses through this “Sprint vs. Marathon” framework, this review will explore how different viruses target key glycolytic enzymes, transporters, and signaling pathways. We will dissect how these divergent strategies contribute to distinct pathogenic outcomes and argue that understanding these unique metabolic hijacking styles is crucial for the development of tailored, host-directed antiviral therapies. This perspective moves beyond simply cataloging metabolic changes, offering a conceptual model to understand the evolutionary logic behind viral metabolic manipulation.

## 2 Key metabolic nodes targeted by viruses: tactics reflecting strategy

### 2.1 HK2

Hexokinase 2 (HK2), which catalyzes the first irreversible step of glycolysis, is a near-universal target for viral manipulation. Yet, the mode and consequence of its activation differ in ways that align with either a “Sprint” or “Marathon” strategy.

For a “Sprinter” like influenza A virus (IAV), infection triggers a rapid increase in HK2 expression (Ren et al., 2021). This is not merely a passive consequence of inflammation. Mechanistically, IAV leverages the PI3K/Akt signaling pathway, which is potently activated by the viral NS1 protein. Specifically, the p85β regulatory subunit of PI3K contains a Src Homology 3 (SH3) domain, which is directly bound by a proline-rich motif on the NS1 protein (Hale et al., 2008). This direct, physical interaction bypasses upstream receptor signaling and robustly activates Akt. Activated Akt, in turn, promotes the nuclear translocation of transcription factors like HIF-1α, which directly binds to the Hypoxia Response Element (HRE) in the HK2 gene promoter, driving its transcription (Ren et al., 2019). This illustrates a direct chain of command: from a specific viral protein domain to a host signaling kinase, and finally to the transcriptional machinery of a key glycolytic enzyme, all geared for rapid metabolic reprogramming.

In contrast, the “Marathon” strategy of Hepatitis B virus (HBV) involves a more indirect, but equally effective, long-term manipulation. The viral HBx protein acts as a transcriptional co-activator. HBx physically interacts with transcription factors like c-Myc and NF-κB (specifically the p65 subunit), enhancing their binding to the HK2 promoter. A key study demonstrated that HBx promotes the phosphorylation of p65 at Serine 536, a critical step for its transcriptional activity, which in turn drives sustained HK2 expression (Chen et al., 2022). This sustained upregulation contributes to the Warburg effect seen in HBV-infected hepatocytes, a metabolic phenotype that supports both viral persistence and long-term oncogenic transformation.

### 2.2 PFK

The key rate-limiting regulator of the glycolytic pathway is 6-phosphoglucose-1 kinase (PFK-1), a tetrameric enzyme consisting of three different subunits, C or P, L, and M, that can form tetramers in both homo- and heterotetrameric forms (Abrantes et al., 2012). Phosphofructokinase (PFK), the next major rate-limiting enzyme, is another critical control point. Its manipulation also showcases strategic divergence. Human cytomegalovirus (HCMV), a classic “Marathoner” that establishes lifelong latency, robustly increases PFK-1 activity (Munger et al., 2006). This activation, mediated via pathways like CaMKK, is not just for producing virions but is also intricately linked to modulating the cellular environment for long-term latency and reactivation, ensuring the virus’s long-term survival within the host (McArdle et al., 2011; Yu et al., 2011). While acute viruses also target PFK, the sophisticated, multi-faceted regulation seen with HCMV underscores a strategy focused on endurance rather than just speed. Interferon-inducible protein 16 (IFI16) down-regulates GLUT4 transcriptional activation by interacting with carbohydrate-responsive element-binding protein (ChREBP), decreasing HCMV-induced lipogenic enzyme transcription, which in turn reduces glucose uptake and consumption, decreases lipid synthesis, and ultimately prevents the formation of new viral particles (Griffante et al., 2022). HBV infection can be modulated by TNF-α After HBV infection, the glucose metabolism of Kupffer cells can be regulated by TNF-α. TNF-α enhances cellular glycolysis by regulating the expression of glycolytic enzymes such as glucokinase (GCK) and phosphofructokinase (PFK) and weakens the antiviral effect of Kupffer cells (Tarasenko et al., 2019). In contrast, Kupffer cells promote the expression of IL-1β, a marker of M1-type macrophages, and decrease the expression of CD163 and IL-10 in M2-type macrophages after HBV infection (Krenkel and Tacke, 2017). The cellular metabolism of HBV-infected Kupffer cells is different from that of conventional M1-type macrophages, exhibiting high oxidative phosphorylation (OXPHOS), which inhibits IL-1β production, and also inhibits HBV replication by suppressing the expression of peroxisome proliferator-activated receptor α (PPARα) and transcription factor Forkhead boxO3 (FOXO3) in macrophages (Li et al., 2022). In summary, PFK, as a key enzyme of glycolysis, plays an important role in the progression of viral infection. Therefore, studies targeting PFK may provide new ideas for the treatment of viral infections.

### 2.3 Glucose transporters (GLUTs): the gatekeepers of glycolysis

While not enzymes themselves, glucose transporters (GLUTs) are the essential gatekeepers that control the influx of glucose into the cell, representing the first critical control point of glycolysis. The choice of which GLUT to upregulate and how can reveal a virus’s strategic priorities. GLUTs are a group of facilitated transporter proteins present in cell membranes for the transport of glucose to the cell membrane (Goyal and Rajala, 2023). Among them, GLUT1 is a widespread glucose transporter protein that is expressed to varying degrees in different cell types, and it is involved in basic glucose uptake in most tissues such as erythrocytes and fibroblasts as well as the brain (Pardridge et al., 1990). Studies have shown that some virally encoded proteins can promote glucose uptake by activating specific signaling pathways and increasing the expression of GLUTs (Muñoz-Pinedo et al., 2012). Efficiently hijacking glycolysis begins with transporting glucose into the cell, making glucose transporters (GLUTs) a primary target. The choice of which GLUT to upregulate and how can reveal the virus’s strategic priorities. Reflecting its “Marathon” strategy, HBV focuses on upregulating GLUT1, the ubiquitous transporter responsible for basal glucose uptake, thereby ensuring a steady, reliable supply of glucose to support its chronic infection (Masson et al., 2017). This is a sustainable, long-term solution. In a more complex maneuver befitting a persistent virus, HCMV has been shown to inhibit GLUT1 but simultaneously induce the translocation of GLUT4 to the cell surface, achieving a net increase in glucose uptake (Yu et al., 2011). This nuanced regulation highlights a sophisticated adaptation for long-term host cell manipulation. The approach of HIV, another chronic virus, also aligns with the “Marathon” style. Elevated GLUT1 expression in HIV-infected CD4++ T cells is crucial not only for providing ATP for reverse transcription but also for maintaining the activated state of the host cell, which is essential for the virus’s life cycle and long-term persistence (Palmer et al., 2014; Loisel-Meyer et al., 2012). The immediate-early protein IE72 encoded by HCMV, on the other hand, inhibits GLUT1 expression in infected cells (Yu et al., 2011), and this inhibition leads to Akt-mediated translocation of GLUT4 to the cell surface, which in turn increases glucose uptake and glycolysis rates (Landini, 1984). IFI16 impedes glucose uptake following HCMV infection by inhibiting the expression of glucose transporter protein GLUT 4, which leads to reduced glucose consumption and a decrease in the number of viral particles (Griffante et al., 2022). In HIV-infected cells, elevated levels of GLUT1 expression in CD4+ T cells contribute to increased glucose transport and increased glycolysis (Palmer et al., 2014). IL-7-induced increase in GLUT1 expression, which also leads to an increase in glucose uptake, has emerged as one of the key factors that make these T-lymphocytes more susceptible to HIV-1 infection (Loisel-Meyer et al., 2012). Upregulation of proinflammatory cytokines in HIV-infected cells, such as IL-1, IL-6, and TNF-α, among others (Palmer and Crowe, 2012), and GLUT1 expression on CD4+ T cells is critical for their proliferation and survival and is regulated by growth and inflammatory cytokines such as IL-2 and IL-7 (Palmer et al., 2016). Increased GLUT1 expression and cellular metabolism may increase the proliferation of HIV stockpile cells and contribute to cellular survival through the provision of ATP substrates for viral DNA replication and of enhance viral proliferation by providing ATP substrates for viral DNA replication and metabolites for cell survival and function (Loftus and Finlay, 2016).

### 2.4 Other enzymes

Glucose-6-phosphate dehydrogenase (G6PD) is the rate-limiting enzyme in the pentose phosphate pathway (PPP) and is responsible for the production of nicotinamide adenine dinucleotide phosphate (NADPH) (Wu et al., 2008). G6PD is elevated in different viral infections, e.g., HBV can drive the upregulation of G6PD expression through its X protein-mediated activation of nuclear factor erythroid 2-related factor 2 (Nrf2) (Liu et al., 2015). HBx can regulate both G6PD and the expression of several genes involved in the gluconeogenesis process (Shin et al., 2011). In addition, upregulation of G6PD expression has been observed in the lung tissues of COVID-19 patients after death (Santos E Silva et al., 2021). The increase in G6PD during viral infection is essential for maintaining NADPH levels, which are subsequently used and depleted by enzymatic and non-enzymatic antioxidant systems to restore the redox homeostatic imbalance caused by viral infection (Yang et al., 2021). In recent years, it has been found that lactate plays an important role in regulating various aspects of T cell proliferation, immune cell metabolism, macrophage polarization, and cytokine production (Manosalva et al., 2021). Lactate production is catalyzed by lactate dehydrogenase (LDH), which controls the interconversion between pyruvate and lactate, a key step in the anaerobic metabolism of glucose. HBV mediates immune escape by converting pyruvate to lactate in a lactate dehydrogenase-dependent manner, inhibiting interferon expression (Wang and Zhang, 2023). In addition, lactate is an activator of HIF-1 α, which can convert cellular glucose metabolism to glycolysis by pretreatment. By stabilizing HIF-1 α, lactate promotes glycolysis in a positive feedback manner, thereby facilitating SARS-CoV-2 infection (Kozlov et al., 2020).

## 3 Signaling pathways: the master control panels of metabolic styles

Viruses do not simply target individual enzymes; they seize control of the master signaling pathways that orchestrate cellular metabolism. The choice of which pathway to dominate and how to modulate it is central to establishing either a “Sprint” or “Marathon” metabolic program. To clarify contrasting strategies, Table 1 summarizes key features of the “Sprint” and “Marathon” metabolic hijacking styles, linking representative viruses to their distinct metabolic and signaling patterns.

**TABLE 1:** A comparative summary of “Sprint” vs. “Marathon” metabolic hijacking styles.

Feature	Sprint style (acute replication)	Marathon style (chronic persistence)
Representative viruses	Influenza A virus (IAV), SARS-CoV-2, respiratory syncytial virus (RSV)	Hepatitis B virus (HBV), human cytomegalovirus (HCMV), HIV, Epstein-Barr virus (EBV)
Primary goal	Maximize viral yield in a short timeframe before immune clearance.	Ensure long-term survival of the host cell to serve as a stable reservoir for persistent viral production.
Metabolic signature	Rapid, robust, and aggressive upregulation of aerobic glycolysis (Warburg effect).	Sustained, nuanced metabolic shift, often coupled with anti-apoptotic signaling and mitochondrial modulation.
Key viral modulators (examples)	IAV NS1 (directly activates PI3K); SARS-CoV-2 Spike/NSPs (broadly interfere with signaling).	HBV HBx (transcriptional co-activator); HCMV pUL38 (disables TSC2); EBV LMP1 (mimics host receptor).
Manipulation of HK2	Rapid transcriptional induction via PI3K/Akt/HIF-1α axis to fuel replicative burst.	Sustained transcriptional upregulation via NF-κB/c-Myc, linked to cell survival and oncogenesis.
Manipulation of PPP (via G6PD)	Primarily for rapid nucleotide synthesis to support fast genome replication (e.g., HIV Vpr).	Primarily for NADPH production to combat chronic oxidative stress and promote cell survival (e.g., HBV HBx/Nrf2).
Mitochondrial strategy	Largely preserved early for biosynthesis, or aggressively targeted to shut down innate immunity (e.g., PB1-F2).	Actively modulated and protected to prevent apoptosis and maintain cellular energy homeostasis.
Key signaling hubs (preferred mechanism)	Direct, potent activation of pathways like PI3K/Akt for immediate, powerful effect.	Nuanced, indirect, or chronic activation of pathways like NF-κB, mTOR to balance replication with cell survival.
Pathological consequence	Acute inflammation, cytokine storm, acute respiratory distress syndrome (ARDS).	Chronic inflammation, fibrosis, immune evasion, and virus-associated cancers.

### 3.1 NF-κB signaling pathway

The nuclear factor kappa-light chain enhancer (NF-kB) is a crucial family of transcription factors that regulate the expression of several genes and have been implicated in a variety of biological processes, including innate and adaptive immunity, inflammation, stress, immune cell development and lymphoid organogenesis (Zhang et al., 2017). Activation of its typical pathway is dependent on phosphorylation and ubiquitination of IkB kinase a/b (IKKa/b) (Qu and Xiao, 2015). Given its central role in linking inflammation to cellular function, it is a primary target for viral manipulation, but it is exploited differently by “Sprinter” and “Marathoner” viruses. For a “Sprinter” like influenza A virus (IAV), aggressive and rapid activation of the NF-κB pathway is a key feature of its strategy. IAV infection activates the NF-κB signaling pathway to promote macrophage glucose metabolism, which plays an important role in the immune response against viral infections and in mediating inflammatory responses (Stifel et al., 2022). Following IAV infection, cytoplasmic I κB α levels are reduced, and phosphorylated I κB α levels are enhanced, implying that NF-κB is activated (Xu and Liu, 2017). HK2 acts as a phospho inhibitor protein kinase, inducing its degradation, which triggers the activation of NF-κB (Guo et al., 2022), and the released NF-κB translocates to the nucleus, where it regulates the transcription of pro-inflammatory mediators, including TNF-α, IL-6, IL-8, and COX-2 (Dai et al., 2011). In addition, methionine enkephalin (MENK), an immune adjuvant, can increase the expression of inflammatory cytokines, activate macrophage antiviral capacity, and ultimately promote changes in glucose metabolism by activating the TLR4-NF-κB- p65 signaling pathway (Tian et al., 2020). Notably, influenza virus surface receptor proteins, such as Mint3, can act as important metabolic regulators, and in macrophages, depletion of Mint3 inhibits the NF-κB signaling pathway by increasing IκBα phosphorylation and AMPK activation and leads to chemokine production (Uematsu et al., 2016). Taken together, the NF-κB signaling pathway has a direct impact on glucose metabolism by regulating influenza A virus receptor expression and macrophage inflammatory mediators. Furthermore, the host’s own inflammatory environment can be co-opted in a “Marathon” strategy. The relationship between Hepatitis B Virus (HBV) infection and the pro-inflammatory cytokine TNF-α is complex and bidirectional. Following HBV infection, the host immune response often leads to the production of TNF-α. This cytokine, in turn, can modulate the metabolic state of liver macrophages (Kupffer cells) by chronically activating the NF-κB pathway, which - promotes a Warburg like metabolism (Tarasenko et al., 2019). This sustained, low-level activation helps create a pro-viral and pro-oncogenic microenvironment that supports the virus’s long-term persistence.

### 3.2 PI3K/Akt/mTOR signaling pathway

The PI3K/Akt pathway exerts much of its control over cell growth and metabolism through its major downstream effector, the mammalian target of rapamycin (mTOR) signaling complex. mTOR, a serine/threonine kinase, is the central component of two distinct complexes, mTORC1 and mTORC2 (Kuss-Duerkop et al., 2017). It is famously the target of the immunosuppressive drug rapamycin, which primarily inhibits mTORC1 activity (Szwed et al., 2021). For a “Sprinter” like IAV, this pathway is a primary weapon. It is exploited for its ability to generate rapid and powerful metabolic shifts, quickly turning on glycolysis, glucose uptake, and protein synthesis to support a burst of virion production (Smallwood et al., 2017). This aggressive, all-in approach prioritizes immediate replication over host cell longevity.

In contrast, viruses executing a “Marathon” strategy modulate this pathway with more finesse. For HCMV, the viral protein pUL38 activates mTORC1 by inhibiting its negative regulator TSC2 (Moorman et al., 2008). This is not just about turning on metabolism; it’s a carefully calibrated move to also block stress-induced apoptosis, thereby ensuring the survival of the infected cell—a prerequisite for a successful long-term infection (Rodríguez-Sánchez et al., 2019). Similarly, HBV infection leads to elevated levels of phosphorylated Akt, which is crucial for both HK2 activation and promoting hepatocyte survival, illustrating a dual-purpose strategy aimed at both replication and persistence (Zhou et al., 2021). In addition, HBV infection of macrophages specifically upregulates the expression of the 6-phosphofructo-2-kinase/fructose-2,6-bisphosphatase (PFKFB3) gene, which causes phagocytic vesicles to aggregate onto actin microfilaments during phagocytosis of macrophages, and enhances glycolysis by regulating the PI3K signaling pathway to provide ATP for actin polymerization (Jiang et al., 2016). In HCMV infection, inhibition of mTORC1 activity effectively prevents HCMV replication in polarized macrophages. UL38 is an early gene of HCMV and an important metabolic regulator. UL38 primarily binds and inhibits tuberous sclerosis protein complex 2 (TSC2) to activate the mTOR pathway (Moorman et al., 2008), which is an inhibitor of mTORC1 signaling (Rodríguez-Sánchez et al., 2019). Thus UL38 protein promotes HCMV virus replication by regulating mTORC1, which in turn induces reprogramming of macrophage glucose metabolism (Bai et al., 2015).

### 3.3 AMPK pathway

The AMPK pathway plays a key role in cellular metabolism by controlling the activity of the mTOR complex, which in turn regulates cellular metabolism. Glycolysis can be up-regulated upon AMPK activation by a variety of mechanisms. AMPK increases glycolytic flux by acting on key enzymes of glycolysis, such as glucose transporter proteins (GLUT1 and GLUT4), HK, and PFK-2 (Fryer et al., 2002). In addition, this pathway interacts extensively with the PI3K-Akt pathway, which is triggered by growth factors (Mihaylova and Shaw, 2011). Activation of AMPK can be achieved by LKB1- or CaMKK-mediated phosphorylation of Thr172 residue (McArdle et al., 2012). Calcium/calmodulin-dependent kinase (CaMKK) is triggered after HCMV infection. Being upstream of the calcium-calmodulin cascade kinase, it will further promote the activation of AMPK kinase and rise in glycolysis. Activated AMPK in turn stimulates glycolysis of GLUT4 (Zhang et al., 2023).

### 3.4 HIF-α

HIF-1 is a transcription factor consisting of HIF-1α and HIF-1β with highly conserved properties of being easily activated in hypoxic environments (O’Carroll and O’Neill, 2021). HIF-1α has been most widely utilized in response to hypoxic environments (Semenza, 2012). It has been found that during viral infection, HIF-1α expression is increased in infected cells, showing that its beneficial effects on the virus far outweigh those on the host (Reyes et al., 2020). Under hypoxic conditions, HIF-1 phosphorylates and inactivates PDH by regulating PDK1, thereby inhibiting the conversion of pyruvate to acetyl coenzyme A and preventing its entry into the tricarboxylic acid cycle (Kim et al., 2006). Hypoxia-inducible factor 1-alpha (HIF-1α) is a master transcription factor for inducing glycolysis and is stabilized by many viruses, serving both “Sprint” and “Marathon” strategies, albeit for slightly different ends. For “Sprinters” like IAV or SARS-CoV-2, rapid stabilization of HIF-1α is a key tactic to quickly switch the cell to a glycolytic state, maximizing ATP production in potentially inflamed and hypoxic microenvironments (Ren et al., 2019; Codo et al., 2020). For “Marathoners” like HBV, the sustained activation of HIF-1α by the HBx protein contributes to the long-term Warburg-like metabolic phenotype of infected hepatocytes, which not only supports viral replication but also promotes angiogenesis and carcinogenesis, pathogenic processes that unfold over years or decades (Moon et al., 2004). HBV protein X (HBx) induces nuclear translocation and transcriptional activation of HIF-1α by activating the MEK1/p42/p44 MAPK pathway (Reyes et al., 2021). SARS-CoV-2 infection often triggers the activation of HIF-1α, which binds to Spike proteins, an important receptor in host cells, triggering the phosphorylation of STAT1, and significantly up-regulating the glycolysis-associated regulator HK2, the CMYC gene, and the HIF-1α transcript levels (Zhou et al., 2020). After SARS-CoV-2 infection of macrophages, a massive increase in the inflammatory factor IL-6 stimulates the interferon α/β response and activation of JAK/STAT signaling, which further enhances the transcription of downstream factors such as HIF-1α (Hadjadj et al., 2020). It has been demonstrated that the HIV-encoded viral protein Vpr controls glucose metabolism in macrophages through the Vpr-HIF-1α axis (Zhao et al., 2021). Vpr is a small molecule multifunctional protein that is essential for HIV replication in macrophages. HIV infection enhances HIF-1α signaling and accelerates pyruvate metabolism, the pentose phosphate pathway, mitochondrial dysfunction, and oxidative stress (Sharifi et al., 2012). Vpr regulates HIF-1α activity and induces increased transcription of enzymes involved in glucose metabolism, such as HK, G6PDH, and PKM2, driving viral replication (Luo et al., 2011). Most of the viruses studied so far can positively regulate this pathway by upregulating the stability of HIF-1α after infection; therefore, an in-depth exploration of how HIF-1 and its associated pathways affect viral infections and disease outcomes could help to identify new therapeutic targets.

### 3.5 Other pathways

**FIGURE 1:**
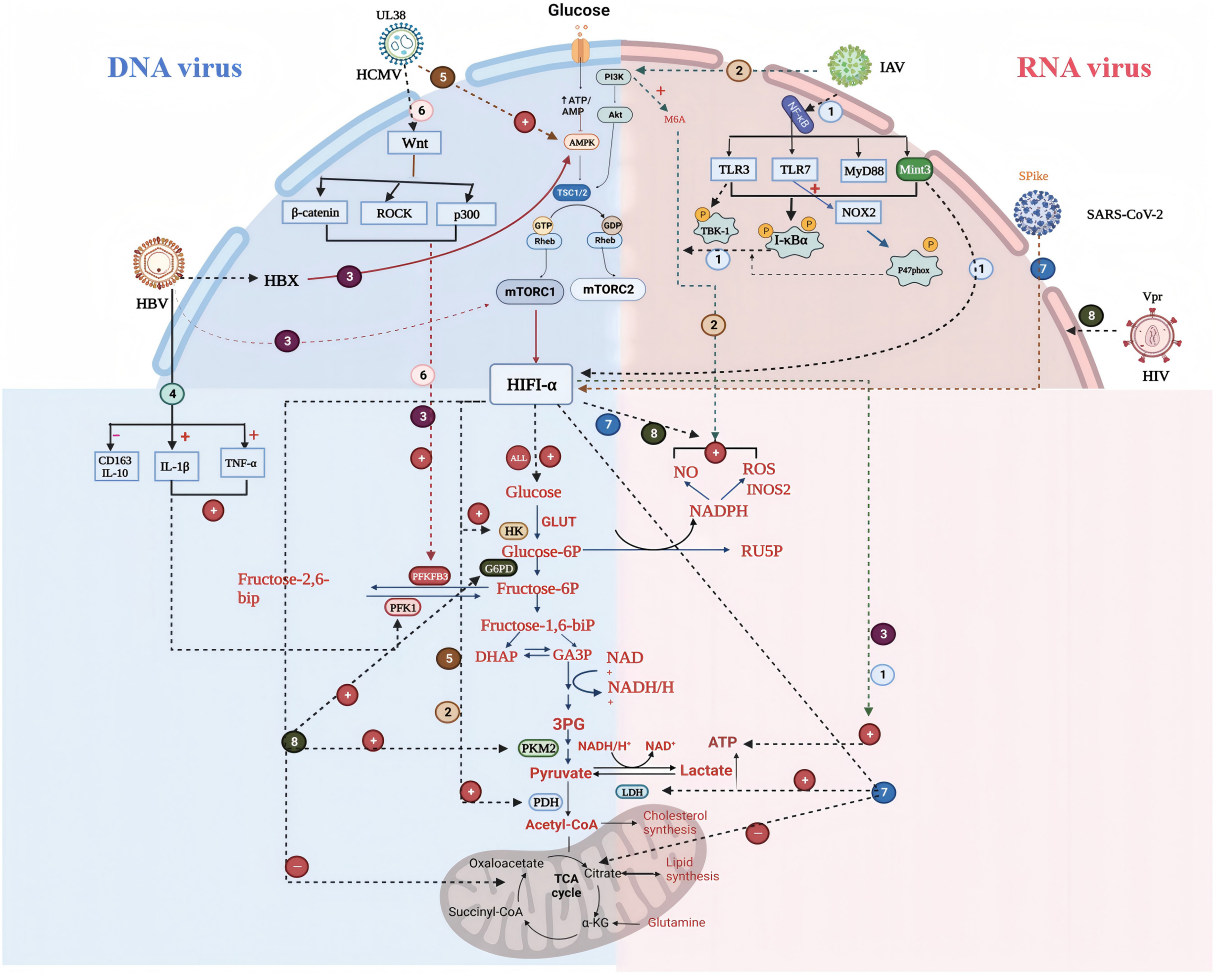
The mechanism of glucose metabolism changes in macrophages caused by common viral infections: ① Influenza A virus (IAV) infects macrophage nuclear factor kappa-light chain enhancer (NF-κB) pathway, inhibits the phosphorylation of regulatory protein IkappaB-alpha (I-κBα), and promotes the increase of glucose metabolites; ② IAV infects macrophage PI3K/mTOR/Akt pathway; ③ HBV infects macrophage PI3K/mTOR/Akt pathway; ④ Macrophages infected with HBV release inflammatory cytokines: IL-1β, TNF-α, IL-10; ⑤ HCMV infects macrophage PI3K/mTOR/Akt pathway; ⑥ HCMV infects macrophage Wnt pathway; ⑦ SARS-CoV-2 infects macrophage HIF1-αpathway; ⑧ HIV infects macrophage HIF1-αpathway; ⊕, promote; ⊖, inhibition; HK, hexokinase; RU5P, ribose 5-phosphate; PKM2, pyruvate kinase; PFK1, phosphofructokinase-1; PDH, pyruvate dehydrogenase; PFKFB3, 6-phosphofructo-2-kinase/fructose-2,6-biphosphatase 2; ALL, all viruses promote glucose production.

Wnt/β-catenin signaling is an important pathway involved in cell cycle control, and differentiation (Angelova et al., 2012). Wnt/β-catenin signaling induces glycolysis to proceed efficiently, which promotes lactate production. When HCMV infection occurs, it affects macrophage metabolic activity by activating the Wnt/β-catenin pathway, which in turn affects macrophage metabolic activity. It has been shown that when HCMV infects the organism resulting in lung infection mainly infects alveolar macrophages, during which HCMV mainly relies on the up-regulation of Wnt signaling, the transcription factor ZEB1, and the zinc finger protein SNAI2; and promotes macrophage glycolysis through the interaction of p300- and CBP/β-catenin and Rho/Rho (ROCK kinase) signaling reprogramming (Kapoor et al., 2013). Kupffer (Kupffer) cells, macrophages derived from bone marrow but residing in the liver, promote the expression of IL-1β, a marker of M1-type macrophages, causing liver injury and decrease the expression of CD163 and IL-10 in M2-type macrophages after HBV infection. The cellular metabolism of HBV-infected Kupffer cells differs from that of conventional M1-type macrophages, exhibiting high oxidative phosphorylation. differently, exhibiting high oxidative phosphorylation (OXPHOS), which inhibits IL-1β production. It also inhibits HBV replication by suppressing the expression of peroxisome proliferator-activated receptor α (PPARα) and the transcription factor Forkhead boxO3 (FOXO3) in macrophages (Krenkel and Tacke, 2017). These mechanisms mentioned above are illustrated in Figure 1, which schematically depicts how influenza virus, HBV, HCMV, SARS-CoV-2, and HIV reprogram macrophage metabolism during infection.

## 4. Antiviral drugs

Research on drugs that target reprogramming of glucose metabolism in viral infections is still ongoing. There are relatively few drugs that directly target reprogramming of glucose metabolism due to viral infections, and some antiviral drugs may indirectly affect glucose metabolism while treating viral infections. For example, influenza virus replication is dependent on host cell glucose in a dose-dependent manner, and treatment of infected cells with glycolysis inhibitors may reduce viral replication (Kohio and Adamson, 2013). Studies have shown that elevated glucose levels and glycolysis promote replication in monocytes infected with SARS-CoV-2 through a HIF-1 α-dependent pathway, and treatment of cells with the glycolysis inhibitor 2-deoxy-d -glucose (2-DG) blocks viral replication (Codo et al., 2020). 2-DG is a synthetic analog of glucose that interferes with glycolysis. In addition, 2-DG inhibits the PPP pathway and glycosylation of S proteins (Chen et al., 2023). Therefore, the drug 2-DG has been used as an antiviral and anti-inflammatory agent to counteract the cytokine storm in COVID-19 patients (Verma et al., 2020). For decades, 2-DG has been used in a variety of applications, including antiviral, anticancer, and antiepileptic. Broad-spectrum antiviral compounds targeting the PI3K/Akt signaling pathway provide host-mediated antiviral responses in a variety of ways and may be considered the best future candidates for the treatment of COVID-19 and related post-COVID syndromes. Although most PI3K inhibitors are used for the treatment of some forms of cancer, their utility in viral infections has not been fully reported (Lekshmi et al., 2023). It has been shown that BEZ235, an inhibitor of PI3K/mTOR, acts as a modulator of viral production following viral infection and inhibits viral replication by decreasing PI3K and mTOR levels, as well as phosphorylation products, blocking transient induction of c-Myc, and decreasing the homeostasis of the pathway for 4E-BP-1 and P85 phosphorylation (Yu et al., 2022). Preclinical studies have reported that atorvastatin downregulates the HBx protein-induced Akt pathway via purinergic receptor (P2X) and further reduces hepatocyte proliferation and invasiveness (Min et al., 2023). mTOR inhibitors, such as sirolimus, everolimus, and tamsulosin, which block downstream signaling of PI3K/Akt, may also play a role in the treatment of herpesvirus infection (Liu and Cohen, 2015). Overall, the research and development of antiviral drugs targeting glycolytic enzymes and pathways in viral infections have provided new options and ideas for the treatment of viral infections (Table 2).

**TABLE 2:** Antiviral agents targeting glycolytic enzymes and signaling pathways.

Drug	Signaling pathways	Glycolytic enzymes regulated	Mechanism	References
2-DG	NF-κB signaling PI3K-AKT-mTOR signaling Hypoxia-inducible factors	Phosphoglucose-isomerase HK2	Targets HK2 and competes with glucose for HK to inhibit glycolysis	Courtney et al., 1973
3-Bromopyruvate	Hypoxia-inducible factors PI3K-AKT-mTOR signaling AMPK	HK2	Directly inhibit HK2 activity	Vital et al., 2022
Metformin	AMPK PI3K-AKT-mTOR signaling	–	Activation of AMP-activated protein kinase	Benedetti et al., 2020
Rapamycin	PI3K-AKT-mTOR signaling	HK2	PI3K/mTOR inhbition	Maiese, 2020
Alkannin/Shikonin	PI3K-AKT-mTOR signaling	PKM2	As the inhibitor of pyruvate kinase M2 (PKM2), inhibited glucose uptake and the production of lactate	Dai et al., 2022
Galloflavin	–	LDH	Lactate dehydrogenase inhibitor	Farabegoli et al., 2012

## 5 Discussion

The evidence reviewed here demonstrates that viral reprogramming of host glucose metabolism is not a uniform process but rather a sophisticated, highly adapted phenomenon. By analyzing the strategies of diverse viruses through the lens of their life cycles, we can discern distinct patterns of metabolic hijacking. Here, we formally propose a conceptual framework that categorizes these patterns into two major styles: a “Sprint” style, optimized for acute, high-yield replication, and a “Marathon” style, engineered for chronic persistence and host co-existence.

### 5.1 The “Sprint” style: a strategy of metabolic “Blitzkrieg”

The “Sprint” or “Blitzkrieg” strategy is characteristic of acute viruses, particularly RNA viruses like influenza A virus (IAV) and SARS-CoV-2. Their primary evolutionary driver is to replicate and transmit to a new host before the adaptive immune response can fully clear the infection. This necessitates a metabolic program that is rapid, robust, and prioritizes speed over efficiency and host cell preservation.

As detailed in the preceding sections, this style involves the aggressive and often direct activation of major signaling hubs like the PI3K/Akt pathway by viral proteins (e.g., IAV’s NS1) (Hale et al., 2008). This triggers a rapid, cascading upregulation of glycolytic flux through key nodes like HK2 and PFK. The stabilization of HIF-1α further amplifies this glycolytic switch (Ren et al., 2019). The metabolic output is channeled toward the massive production of ATP and biosynthetic precursors (nucleotides, amino acids, lipids) needed for an explosive burst of virion assembly. The consequence of this metabolic violence is often a powerful pro-inflammatory response, or “cytokine storm,” which is itself metabolically demanding and fueled by the very glycolytic shift the virus initiated (Tannahill et al., 2013). For a “Sprinter,” the collateral damage to the host is a secondary concern to the primary goal of rapid propagation.

### 5.2 The “Marathon” style: a strategy of metabolic “Symbiosis” and endurance

In stark contrast, the “Marathon” style is the hallmark of viruses that establish chronic, latent, or persistent infections, such as the DNA viruses HBV and HCMV, and the retrovirus HIV. Their long-term success depends on a fundamentally different principle: the preservation of the host cell as a stable, long-term factory for viral production. This requires a metabolic hijacking that is subtle, sustainable, and multi-faceted.

“Marathoners” also upregulate glycolysis, but their methods and goals are more nuanced. The activation of GLUTs, HK2, and PFK is often more sustained and integrated with cellular survival signals (Munger et al., 2006; Palmer et al., 2014). The modulation of the PI3K/Akt/mTOR pathway by these viruses, for instance, is not just about turning on biosynthesis; it is critically coupled with inhibiting apoptosis. The HCMV pUL38 protein’s function is a prime example of this dual strategy, ensuring the cell stays alive under stress (Moorman et al., 2008). Furthermore, “Marathoners” often engage in more complex mitochondrial manipulation. Instead of simply draining the TCA cycle for precursors, they may actively modulate mitochondrial dynamics and bioenergetics to prevent the induction of cell death, thereby securing their replicative niche for months, years, or even a lifetime (Wang and Zhang, 2023). This co-opting of host survival pathways is the essence of the “Marathon” strategy, representing a sophisticated form of metabolic co-existence, albeit a pathogenic one.

### 5.3 Evolutionary rationale and pathological implications

The divergence into “Sprint” and “Marathon” styles is not accidental but is deeply rooted in viral evolution. An acute RNA virus with a high mutation rate, like IAV, benefits from a rapid life cycle that maximizes transmission opportunities before host immunity adapts. A large, slow-replicating DNA virus, like HCMV, has a greater investment in each infected cell and benefits from a strategy that ensures the host cell’s longevity.

This framework also provides a powerful lens for understanding viral pathogenesis. The “Sprint” style’s aggressive metabolic reprogramming is intrinsically linked to acute, inflammation-driven pathology like ARDS. The “Marathon” style, with its focus on sustained cell survival and proliferation, is directly linked to long-term pathologies such as chronic inflammation, fibrosis, and virus-associated cancers, like HBV-induced hepatocellular carcinoma (Chen et al., 2022).

### 5.4 Therapeutic implications: from a one-size-fits-all to a style-specific approach

Recognizing these distinct metabolic styles has profound implications for developing host-directed antiviral therapies. A broad-spectrum glycolysis inhibitor like 2-DG might be effective against “Sprinters” by cutting off their immediate fuel supply (Codo et al., 2020). For example, early in the COVID-19 pandemic, pioneering work by Bojkova et al. (2020) demonstrated that the glucose analog 2-deoxy-D-glucose (2-DG) could effectively inhibit SARS-CoV-2 replication in cell culture. Subsequent studies, including those by Codo et al. (2020) further confirmed that SARS-CoV-2 replication is highly dependent on a HIF-1α-driven glycolytic state, reinforcing the therapeutic potential of targeting this metabolic vulnerability. However, for “Marathoners,” a more effective strategy might involve targeting the pathways that link metabolism to cell survival. For example, drugs that modulate mTOR signaling or restore apoptotic sensitivity in infected cells could be uniquely effective against chronic infections like HCMV or HBV, by dismantling the stable replicative niche these viruses have so carefully constructed. This “style-specific” therapeutic approach represents a more sophisticated future for metabolism-targeting antiviral strategies.

## 6 Conclusion and future perspectives

### 6.1 Conclusion

In conclusion viral infections profoundly reprogram host cellular glucose metabolism, a process essential for their replication and survival. However, this review has moved beyond this general observation to propose that these metabolic manipulations are not random but follow distinct, evolutionarily conserved strategies. By introducing a “Sprint vs. Marathon” conceptual framework, we have argued that the style of metabolic hijacking is intrinsically linked to a virus’s life cycle and pathogenic potential. Acute ‘Sprinters” like IAV employ a rapid and aggressive metabolic takeover to fuel an explosive replicative burst, while chronic “Marathoners” like HBV and HCMV orchestrate a more subtle and sustainable reprogramming to ensure long-term host cell survival and persistence.

This comparative model provides a new lens through which to interpret the vast and complex data on virus-host interactions. It recasts metabolic pathways not merely as a set of resources to be plundered, but as a strategic battleground where the “rules of engagement” are dictated by the virus’s fundamental biology. The evidence reviewed here, from the differential activation of key enzymes like HK2 to the nuanced modulation of master signaling pathways like PI3K/Akt/mTOR, strongly supports this strategic dichotomy. Understanding whether a virus is built for a short, violent sprint or a long, grueling marathon is key to understanding the disease it causes.

### 6.2 Future perspectives

Looking forward, this framework opens up several exciting and critical avenues for future research:

Direct “Head-to-Head” experimental validation: There is a pressing need for direct, side-by-side experimental comparisons of “Sprinter” and “Marathoner” viruses in the same cellular system, using multi-omics approaches to provide definitive evidence for the proposed metabolic signatures.

Unraveling the metabolism of viral variants: A key unanswered question is whether different viral variants (e.g., ancestral SARS-CoV-2 vs. Omicron) exhibit different metabolic hijacking styles and whether this correlates with altered pathogenicity.

*In vivo* and tissue-specific metabolic mapping: Advanced techniques like imaging mass cytometry and spatial transcriptomics are needed to map the metabolic landscape of infected tissues, revealing how viruses reprogram different immune cell populations in their native microenvironments.

Developing “Style-Specific” therapeutics: The ultimate goal is to translate this knowledge into better treatments. Future research should focus on designing and testing host-directed therapies that are tailored to a virus’s specific metabolic style, moving beyond a one-size-fits-all approach.

By pursuing these directions, the field can move towards a deeper, more predictive understanding of viral pathogenesis, paving the way for a new generation of smarter, more targeted antiviral strategies.

## References

[B1] Abrantes J. L. Alves C. M. Costa J. Almeida F. C. L. Sola-Penna M. Fontes C. F. L. (2012). Herpes simplex type 1 activates glycolysis through engagement of the enzyme 6-phosphofructo-1-kinase (PFK-1). *Biochim. Biophys. Acta* 1822 1198–1206. 10.1016/j.bbadis.2012.04.011 22542512

[B2] Angelova M. Zwezdaryk K. Ferris M. Shan B. Morris C. A. Sullivan D. E. (2012). Human cytomegalovirus infection dysregulates the canonical Wnt/β-catenin signaling pathway. *PLoS Pathog.* 8:e1002959. 10.1371/journal.ppat.1002959 23071438 PMC3469659

[B3] Bai Y. Xuan B. Liu H. Zhong J. Yu D. Qian Z. (2015). Tuberous sclerosis complex protein 2-independent activation of mTORC1 by human cytomegalovirus pUL38. *J. Virol.* 89 7625–7635. 10.1128/JVI.01027-15 25972538 PMC4505643

[B4] Benedetti F. Sorrenti V. Buriani A. Fortinguerra S. Scapagnini G. Zella D. (2020). Resveratrol, rapamycin and metformin as modulators of antiviral pathways. *Viruses* 12:1458. 10.3390/v12121458 33348714 PMC7766714

[B5] Bojkova D. Klann K. Koch B. Widera M. Krause D. Ciesek S. (2020). Proteomics of SARS-CoV-2-infected host cells reveals therapy targets. *Nature* 583 469–472. 10.1038/s41586-020-2332-7 32408336 PMC7616921

[B6] Chen L. Lin X. Lei Y. Xu X. Zhou Q. Chen Y. (2022). Aerobic glycolysis enhances HBx-initiated hepatocellular carcinogenesis via NF-κBp65/HK2 signalling. *J. Exp. Clin. Cancer Res.* 41:329. 10.1186/s13046-022-02531-x 36411480 PMC9677649

[B7] Chen P. Wu M. He Y. Jiang B. He M.-L. (2023). Metabolic alterations upon SARS-CoV-2 infection and potential therapeutic targets against coronavirus infection. *Signal Transduct. Target. Ther.* 8:237. 10.1038/s41392-023-01510-8 37286535 PMC10244875

[B8] Codo A. C. Davanzo G. G. Monteiro L. de B. de Souza G. F. Muraro S. P. (2020). Elevated glucose levels favor SARS-CoV-2 infection and monocyte response through a HIF-1α/glycolysis-dependent axis. *Cell Metab* 32:437–446.e5. 10.1016/j.cmet.2020.07.007 32697943 PMC7367032

[B9] Courtney R. J. Steiner S. M. Benyesh-Melnick M. (1973). Effects of 2-deoxy-D-glucose on herpes simplex virus replication. *Virology* 52 447–455. 10.1016/0042-6822(73)90340-1 4350224

[B10] Dai X. Zhang L. Hong T. (2011). Host cellular signaling induced by influenza virus. *Sci. China Life Sci.* 54 68–74. 10.1007/s11427-010-4116-z 21253874

[B11] Dai Y. Liu Y. Li J. Jin M. Yang H. Huang G. (2022). Shikonin inhibited glycolysis and sensitized cisplatin treatment in non-small cell lung cancer cells via the exosomal pyruvate kinase M2 pathway. *Bioengineered* 13 13906–13918. 10.1080/21655979.2022.2086378 35706397 PMC9275963

[B12] Farabegoli F. Vettraino M. Manerba M. Fiume L. Roberti M. Di Stefano G. (2012). Galloflavin, a new lactate dehydrogenase inhibitor, induces the death of human breast cancer cells with different glycolytic attitude by affecting distinct signaling pathways. *Eur. J. Pharm. Sci.* 47 729–738. 10.1016/j.ejps.2012.08.012 22954722

[B13] Fryer L. G. D. Parbu-Patel A. Carling D. (2002). Protein kinase inhibitors block the stimulation of the AMP-activated protein kinase by 5-amino-4-imidazolecarboxamide riboside. *FEBS Lett.* 531 189–192. 10.1016/s0014-5793(02)03501-9 12417310

[B14] Girdhar K. Powis A. Raisingani A. Chrudinová M. Huang R. Tran T. (2021). Viruses and metabolism: The effects of viral infections and viral insulins on host metabolism. *Annu. Rev. Virol.* 8 373–391. 10.1146/annurev-virology-091919-102416 34586876 PMC9175272

[B15] Goyal P. Rajala M. S. (2023). Reprogramming of glucose metabolism in virus infected cells. *Mol. Cell Biochem.* 478 2409–2418. 10.1007/s11010-023-04669-4 36709223 PMC9884135

[B16] Griffante G. Hewelt-Belka W. Albano C. Gugliesi F. Pasquero S. Castillo Pacheco S. F. (2022). IFI16 impacts metabolic reprogramming during human cytomegalovirus infection. *mBio* 13:e0043522. 10.1128/mbio.00435-22 35420480 PMC9239058

[B17] Guo D. Tong Y. Jiang X. Meng Y. Jiang H. Du L. (2022). Aerobic glycolysis promotes tumor immune evasion by hexokinase2-mediated phosphorylation of IκBα. *Cell Metab.* 34:1312–1324.e6. 10.1016/j.cmet.2022.08.002 36007522

[B18] Hadjadj J. Yatim N. Barnabei L. Corneau A. Boussier J. Smith N. (2020). Impaired type I interferon activity and inflammatory responses in severe COVID-19 patients. *Science* 369 718–724. 10.1126/science.abc6027 32661059 PMC7402632

[B19] Hale B. G. Randall R. E. Ortín J. Jackson D. (2008). The multifunctional NS1 protein of influenza A viruses. *J. Gen. Virol.* 89, 2359–2376. 10.1099/vir.0.2008/004606-0 18796704

[B20] Jiang H. Shi H. Sun M. Wang Y. Meng Q. Guo P. (2016). PFKFB3-driven macrophage glycolytic metabolism is a crucial component of innate antiviral defense. *J. Immunol.* 197 2880–2890. 10.4049/jimmunol.1600474 27566823

[B21] Kapoor A. He R. Venkatadri R. Forman M. Arav-Boger R. (2013). Wnt modulating agents inhibit human cytomegalovirus replication. *Antimicrob. Agents Chemother.* 57 2761–2767. 10.1128/AAC.00029-13 23571549 PMC3716142

[B22] Kim J. Tchernyshyov I. Semenza G. L. Dang C. V. (2006). HIF-1-mediated expression of pyruvate dehydrogenase kinase: A metabolic switch required for cellular adaptation to hypoxia. *Cell Metab.* 3 177–185. 10.1016/j.cmet.2006.02.002 16517405

[B23] Kohio H. P. Adamson A. L. (2013). Glycolytic control of vacuolar-type ATPase activity: A mechanism to regulate influenza viral infection. *Virology* 444 301–309. 10.1016/j.virol.2013.06.026 23876457

[B24] Kozlov A. M. Lone A. Betts D. H. Cumming R. C. (2020). Lactate preconditioning promotes a HIF-1α-mediated metabolic shift from OXPHOS to glycolysis in normal human diploid fibroblasts. *Sci. Rep.* 10:8388. 10.1038/s41598-020-65193-9 32433492 PMC7239882

[B25] Krenkel O. Tacke F. (2017). Liver macrophages in tissue homeostasis and disease. *Nat. Rev. Immunol.* 17 306–321. 10.1038/nri.2017.11 28317925

[B26] Kuss-Duerkop S. K. Wang J. Mena I. White K. Metreveli G. Sakthivel R. (2017). Influenza virus differentially activates mTORC1 and mTORC2 signaling to maximize late stage replication. *PLoS Pathog.* 13:e1006635. 10.1371/journal.ppat.1006635 28953980 PMC5617226

[B27] Landini M. P. (1984). Early enhanced glucose uptake in human cytomegalovirus-infected cells. *J. Gen. Virol.* 65 1229–1232. 10.1099/0022-1317-65-7-1229 6086816

[B28] Lekshmi V. S. Asha K. Sanicas M. Asi A. Arya U. M. Kumar B. (2023). PI3K/Akt/Nrf2 mediated cellular signaling and virus-host interactions: Latest updates on the potential therapeutic management of SARS-CoV-2 infection. *Front. Mol. Biosci.* 10:1158133. 10.3389/fmolb.2023.1158133 37325475 PMC10267462

[B29] Li Y. Zhu Y. Feng S. Ishida Y. Chiu T.-P. Saito T. (2022). Macrophages activated by hepatitis B virus have distinct metabolic profiles and suppress the virus via IL-1β to downregulate PPARα and FOXO3. *Cell Rep.* 38:110284. 10.1016/j.celrep.2021.110284 35081341 PMC8830375

[B30] Liu B. Fang M. He Z. Cui D. Jia S. Lin X. (2015). Hepatitis B virus stimulates G6PD expression through HBx-mediated Nrf2 activation. *Cell Death Dis.* 6:e1980. 10.1038/cddis.2015.322 26583321 PMC4670929

[B31] Liu X. Cohen J. I. (2015). The role of PI3K/Akt in human herpesvirus infection: From the bench to the bedside. *Virology* 479–480 568–577. 10.1016/j.virol.2015.02.040 25798530 PMC4424147

[B32] Loftus R. M. Finlay D. K. (2016). Immunometabolism: Cellular metabolism turns immune regulator. *J. Biol. Chem.* 291 1–10. 10.1074/jbc.R115.693903 26534957 PMC4697146

[B33] Loisel-Meyer S. Swainson L. Craveiro M. Oburoglu L. Mongellaz C. Costa C. (2012). Glut1-mediated glucose transport regulates HIV infection. *Proc. Natl. Acad. Sci. U.S.A.* 109 2549–2554. 10.1073/pnas.1121427109 22308487 PMC3289356

[B34] Luo W. Hu H. Chang R. Zhong J. Knabel M. O’Meally R. (2011). Pyruvate kinase M2 is a PHD3-stimulated coactivator for hypoxia-inducible factor 1. *Cell* 145 732–744. 10.1016/j.cell.2011.03.054 21620138 PMC3130564

[B35] Maiese K. (2020). The mechanistic target of rapamycin (mTOR): Novel considerations as an antiviral treatment. *Curr. Neurovasc. Res.* 17 332–337. 10.2174/1567202617666200425205122 32334502 PMC7541431

[B36] Manosalva C. Quiroga J. Hidalgo A. I. Alarcón P. Anseoleaga N. Hidalgo M. A. (2021). Role of lactate in inflammatory processes: Friend or foe. *Front. Immunol.* 12:808799. 10.3389/fimmu.2021.808799 35095895 PMC8795514

[B37] Masson J. J. Billings H. W. Palmer C. S. (2017). Metabolic reprogramming during hepatitis B disease progression offers novel diagnostic and therapeutic opportunities. *Antivir. Chem. Chemother.* 25 53–57. 10.1177/2040206617701372 28768434 PMC5890528

[B38] McArdle J. Moorman N. J. Munger J. (2012). HCMV targets the metabolic stress response through activation of AMPK whose activity is important for viral replication. *PLoS Pathog.* 8:e1002502. 10.1371/journal.ppat.1002502 22291597 PMC3266935

[B39] McArdle J. Schafer X. L. Munger J. (2011). Inhibition of calmodulin-dependent kinase kinase blocks human cytomegalovirus-induced glycolytic activation and severely attenuates production of viral progeny. *J. Virol.* 85 705–714. 10.1128/JVI.01557-10 21084482 PMC3019999

[B40] Mihaylova M. M. Shaw R. J. (2011). The AMPK signalling pathway coordinates cell growth, autophagy and metabolism. *Nat. Cell Biol.* 13 1016–1023. 10.1038/ncb2329 21892142 PMC3249400

[B41] Min Y. Wei X. Xia X. Wei Z. Li R. Jin J. (2023). Hepatitis B virus infection: An insight into the clinical connection and molecular interaction between hepatitis B virus and host extrahepatic cancer risk. *Front. Immunol.* 14:1141956. 10.3389/fimmu.2023.1141956 36936956 PMC10014788

[B42] Moon E.-J. Jeong C.-H. Jeong J.-W. Kim K. R. Yu D.-Y. Murakami S. (2004). Hepatitis B virus X protein induces angiogenesis by stabilizing hypoxia-inducible factor-1alpha. *FASEB J.* 18 382–384. 10.1096/fj.03-0153fje 14688211

[B43] Moorman N. J. Cristea I. M. Terhune S. S. Rout M. P. Chait B. T. Shenk T. (2008). Human cytomegalovirus protein UL38 inhibits host cell stress responses by antagonizing the tuberous sclerosis protein complex. *Cell Host Microbe* 3 253–262. 10.1016/j.chom.2008.03.002 18407068 PMC2759192

[B44] Munger J. Bajad S. U. Coller H. A. Shenk T. Rabinowitz J. D. (2006). Dynamics of the cellular metabolome during human cytomegalovirus infection. *PLoS Pathog.* 2:e132. 10.1371/journal.ppat.0020132 17173481 PMC1698944

[B45] Muñoz-Pinedo C. El Mjiyad N. Ricci J.-E. (2012). Cancer metabolism: Current perspectives and future directions. *Cell Death Dis.* 3:e248. 10.1038/cddis.2011.123 22237205 PMC3270265

[B46] O’Carroll S. M. O’Neill L. A. J. (2021). Targeting immunometabolism to treat COVID-19. *Immunother. Adv.* 1:ltab013. 10.1093/immadv/ltab013 34240083 PMC8195165

[B47] Palmer C. S. Crowe S. M. (2012). The role of glucose and lipid metabolism in the pathogenesis of HIV-1 infection. *Curr. Trends Immunol.* 13 37–50.

[B48] Palmer C. S. Cherry C. L. Sada-Ovalle I. Singh A. Crowe S. M. (2016). Glucose metabolism in T cells and monocytes: New perspectives in HIV pathogenesis. *EBioMedicine* 6 31–41. 10.1016/j.ebiom.2016.02.012 27211546 PMC4856752

[B49] Palmer C. S. Ostrowski M. Gouillou M. Tsai L. Yu D. Zhou J. (2014). Increased glucose metabolic activity is associated with CD4+ T-cell activation and depletion during chronic HIV infection. *AIDS* 28 297–309. 10.1097/QAD.0000000000000128 24335483 PMC4293200

[B50] Pant A. Dsouza L. Yang Z. (2021). Alteration in cellular signaling and metabolic reprogramming during viral infection. *mBio* 12:e0063521. 10.1128/mBio.00635-21 34517756 PMC8546648

[B51] Pardridge W. M. Boado R. J. Farrell C. R. (1990). Brain-type glucose transporter (GLUT-1) is selectively localized to the blood-brain barrier. Studies with quantitative western blotting and in situ hybridization. *J. Biol. Chem.* 265 18035–18040. 2211679

[B52] Qu Z. Xiao G. (2015). Systematic detection of noncanonical NF-κB activation. *Methods Mol. Biol.* 1280 121–154. 10.1007/978-1-4939-2422-6_8 25736747

[B53] Ren L. Zhang W. Han P. Zhang J. Zhu Y. Meng X. (2019). Influenza A virus (H1N1) triggers a hypoxic response by stabilizing hypoxia-inducible factor-1α via inhibition of proteasome. *Virology* 530 51–58. 10.1016/j.virol.2019.02.010 30780125

[B54] Ren L. Zhang W. Zhang J. Zhang J. Zhang H. Zhu Y. (2021). Influenza A virus (H1N1) infection induces glycolysis to facilitate viral replication. *Virol. Sin.* 36 1532–1542. 10.1007/s12250-021-00433-4 34519916 PMC8692537

[B55] Reyes A. Corrales N. Gálvez N. M. S. Bueno S. M. Kalergis A. M. González P. A. (2020). Contribution of hypoxia inducible factor-1 during viral infections. *Virulence* 11 1482–1500. 10.1080/21505594.2020.1836904 33135539 PMC7605355

[B56] Reyes A. Duarte L. F. Farías M. A. Tognarelli E. Kalergis A. M. Bueno S. M. (2021). Impact of hypoxia over human viral infections and key cellular processes. *Int. J. Mol. Sci.* 22:7954. 10.3390/ijms22157954 34360716 PMC8347150

[B57] Rodríguez-Sánchez I. Schafer X. L. Monaghan M. Munger J. (2019). The human cytomegalovirus UL38 protein drives mTOR-independent metabolic flux reprogramming by inhibiting TSC2. *PLoS Pathog.* 15:e1007569. 10.1371/journal.ppat.1007569 30677091 PMC6363234

[B58] Santos E Silva J. C. Vasconcelos A. P. Noma I. H. Y. Noronha N. Y. Aquino R. (2021). Gene signatures of autopsy lungs from obese patients with COVID-19. *Clin. Nutr. ESPEN* 44 475–478. 10.1016/j.clnesp.2021.05.004 34330510 PMC8149170

[B59] Semenza G. L. (2012). Hypoxia-inducible factors in physiology and medicine. *Cell* 148 399–408. 10.1016/j.cell.2012.01.021 22304911 PMC3437543

[B60] Sharifi H. J. Furuya A. M. de Noronha C. M. C. (2012). The role of HIV-1 Vpr in promoting the infection of nondividing cells and in cell cycle arrest. *Curr. Opin. HIV AIDS* 7 187–194. 10.1097/COH.0b013e32835049e0 22274659 PMC3802534

[B61] Shin H.-J. Park Y.-H. Kim S.-U. Moon H.-B. Park D. S. Han Y.-H. (2011). Hepatitis B virus X protein regulates hepatic glucose homeostasis via activation of inducible nitric oxide synthase. *J. Biol. Chem.* 286 29872–29881. 10.1074/jbc.M111.259978 21690090 PMC3191028

[B62] Smallwood H. S. Duan S. Morfouace M. Rezinciuc S. Shulkin B. L. Shelat A. (2017). Targeting metabolic reprogramming by influenza infection for therapeutic intervention. *Cell Rep.* 19 1640–1653. 10.1016/j.celrep.2017.04.039 28538182 PMC5599215

[B63] Stifel U. Caratti G. Tuckermann J. (2022). Novel insights into the regulation of cellular catabolic metabolism in macrophages through nuclear receptors. *FEBS Lett.* 596 2617–2629. 10.1002/1873-3468.14474 35997656

[B64] Szwed A. Kim E. Jacinto E. (2021). Regulation and metabolic functions of mTORC1 and mTORC2. *Physiol. Rev.* 101 1371–1426. 10.1152/physrev.00026.2020 33599151 PMC8424549

[B65] Tannahill G. M. Curtis A. M. Adamik J. Palsson-McDermott E. M. McGettrick A. F. Goel G. (2013). Succinate is an inflammatory signal that induces IL-1β through HIF-1α. *Nature* 496, 238–242. 10.1038/nature11986 23535595 PMC4031686

[B66] Tarasenko T. N. Jestin M. Matsumoto S. Saito K. Hwang S. Gavrilova O. (2019). Macrophage derived TNFα promotes hepatic reprogramming to Warburg-like metabolism. *J. Mol. Med.* 97 1231–1243. 10.1007/s00109-019-01786-w 31053970 PMC6715514

[B67] Thyrsted J. Holm C. K. (2021). Virus-induced metabolic reprogramming and innate sensing hereof by the infected host. *Curr. Opin. Biotechnol.* 68 44–50. 10.1016/j.copbio.2020.10.004 33113498

[B68] Tian J. Qu N. Jiao X. Wang X. Geng J. Griffin N. (2020). Methionine enkephalin inhibits influenza A virus infection through upregulating antiviral state in RAW264.7 cells. *Int. Immunopharmacol.* 78:106032. 10.1016/j.intimp.2019.106032 31835089

[B69] Uematsu T. Fujita T. Nakaoka H. J. Hara T. Kobayashi N. Murakami Y. (2016). Mint3/Apba3 depletion ameliorates severe murine influenza pneumonia and macrophage cytokine production in response to the influenza virus. *Sci. Rep.* 6:37815. 10.1038/srep37815 27883071 PMC5121658

[B70] Verma A. Adhikary A. Woloschak G. Dwarakanath B. S. Papineni R. V. L. (2020). A combinatorial approach of a polypharmacological adjuvant 2-deoxy-D-glucose with low dose radiation therapy to quell the cytokine storm in COVID-19 management. *Int. J. Radiat. Biol.* 96 1323–1328. 10.1080/09553002.2020.1818865 32910699

[B71] Vital P. da S. Bonatelli M. Dias M. P. de Salis L. V. V. Pinto M. T. (2022). 3-Bromopyruvate suppresses the malignant phenotype of vemurafenib-resistant melanoma cells. *Int. J. Mol. Sci.* 23:15650. 10.3390/ijms232415650 36555289 PMC9779063

[B72] Wang H. Zhang J. (2023). The glucose metabolic reprogramming in hepatitis B virus infection and hepatitis B virus associated diseases. *J. Gastroenterol. Hepatol.* 38 1886–1891. 10.1111/jgh.16340 37654246

[B73] Wu Y.-H. Tseng C.-P. Cheng M.-L. Ho H.-Y. Shih S.-R. Chiu D. T.-Y. (2008). Glucose-6-phosphate dehydrogenase deficiency enhances human coronavirus 229E infection. *J. Infect. Dis.* 197 812–816. 10.1086/528377 18269318 PMC7199897

[B74] Xu Y. Liu L. (2017). Curcumin alleviates macrophage activation and lung inflammation induced by influenza virus infection through inhibiting the NF-κB signaling pathway. *Influenza Other Respir. Viruses* 11 457–463. 10.1111/irv.12459 28646616 PMC5596526

[B75] Yang H.-C. Ma T.-H. Tjong W.-Y. Stern A. Chiu D. T.-Y. (2021). G6PD deficiency, redox homeostasis, and viral infections: Implications for SARS-CoV-2 (COVID-19). *Free Radic. Res.* 55 364–374. 10.1080/10715762.2020.1866757 33401987 PMC7799378

[B76] Yu S. Ge H. Li S. Qiu H.-J. (2022). Modulation of macrophage polarization by viruses: Turning Off/On host antiviral responses. *Front. Microbiol.* 13:839585. 10.3389/fmicb.2022.839585 35222345 PMC8874017

[B77] Yu Y. Maguire T. G. Alwine J. C. (2011). Human cytomegalovirus activates glucose transporter 4 expression to increase glucose uptake during infection. *J. Virol.* 85 1573–1580. 10.1128/JVI.01967-10 21147915 PMC3028904

[B78] Zhang P. Pan S. Yuan S. Shang Y. Shu H. (2023). Abnormal glucose metabolism in virus associated sepsis. *Front. Cell Infect. Microbiol.* 13:1120769. 10.3389/fcimb.2023.1120769 37124033 PMC10130199

[B79] Zhang Q. Lenardo M. J. Baltimore D. (2017). 30 Years of NF-κB: A blossoming of relevance to human pathobiology. *Cell* 168 37–57. 10.1016/j.cell.2016.12.012 28086098 PMC5268070

[B80] Zhao L. Wang S. Xu M. He Y. Zhang X. Xiong Y. (2021). Vpr counteracts the restriction of LAPTM5 to promote HIV-1 infection in macrophages. *Nat. Commun.* 12:3691. 10.1038/s41467-021-24087-8 34140527 PMC8211709

[B81] Zhou F. Yu T. Du R. Fan G. Liu Y. Liu Z. (2020). Clinical course and risk factors for mortality of adult inpatients with COVID-19 in Wuhan, China: A retrospective cohort study. *Lancet* 395 1054–1062. 10.1016/S0140-6736(20)30566-3 32171076 PMC7270627

[B82] Zhou L. He R. Fang P. Li M. Yu H. Wang Q. (2021). Hepatitis B virus rigs the cellular metabolome to avoid innate immune recognition. *Nat. Commun.* 12:98. 10.1038/s41467-020-20316-8 33397935 PMC7782485

